# Multiclass Comparative Analysis of Veterinary Drugs, Mycotoxins, and Pesticides in Bovine Milk by Ultrahigh-Performance Liquid Chromatography–Hybrid Quadrupole–Linear Ion Trap Mass Spectrometry

**DOI:** 10.3390/foods11030331

**Published:** 2022-01-25

**Authors:** Qi Jia, Jing Qiu, Lin Zhang, Guangqin Liao, Yanbo Jia, Yongzhong Qian

**Affiliations:** 1 Institute of Quality Standards and Testing Technology for Agro-Products, Chinese Academy of Agricultural Sciences, Beijing 100081, China; jiaqi@caas.cn (Q.J.); qiujing@caas.cn (J.Q.); lindeliner@foxmail.com (L.Z.); lgqlightcheng@163.com (G.L.); 2 Supervision and Inspection Center of Quality and Safety for Agro-Products, Ministry of Agriculture and Rural Affairs, Beijing 100081, China; 3 Shanghai AB Sciex Analytical Instrument Trading Co., Ltd., Beijing 100015, China; yanbo.jia@sciex.com

**Keywords:** milk, veterinary drug, mycotoxin, pesticide, liquid chromatography–triple qtrap mass spectrometry

## Abstract

A multiclass and multiresidue method for simultaneously screening and confirming veterinary drugs, mycotoxins, and pesticides in bovine milk was developed and validated with ultrahigh-performance liquid chromatography–hybrid quadrupole–linear ion trap mass spectrometry (UHPLC-Qtrap-MS). A total of 209 targeted contaminants were effectively extracted using an optimized QuEChERS method. Quantitative and qualitative confirmation were achieved simultaneously by multiple reaction monitoring–information-dependent acquisition–enhanced product ion (MRM-IDA-EPI) scan mode. The validation results exhibited a good sensitivity with the LOQs of 0.05–5 μg/kg, which was satisfactory for their MRLs in China or EU. The recoveries of in-house spiked samples were in the range of 51.20–129.76% with relative standard deviations (RSD) between replicates (*n* = 3) 0.82% and 19.76%. The test results of 140 milk samples from supermarkets and dairy farms in China showed that cloxacillin, aflatoxin M1, acetamiprid, and fipronil sulfone were found with lower concentrations. Combined with the residue results from the literature, penicillin G and cloxacillin (beta-lactams), enrofloxacin and ciprofloxacin (fluoroquinolones), and sulfamerazine (sulfonamides) were more frequently detected in different countries and need to receive more attention regarding their monitoring and control.

## 1. Introduction

The quality and safety of bovine milk is an important issue for consumers in the world because it is an important resource of lipids, proteins, amino acids, vitamins, and minerals [1]. However, many contaminants would produce residues in bovine milk because of nonstandard or illegal direct application and migration from feed and the environment [2]. A wide variety of veterinary drugs including beta-lactams (penicillins [3,4] and cephalosporin [5]), fluoroquinolones [5,6,7], sulfonamides [7], and tetracycline [8] have been used directly to prevent and control cow diseases such as mastitis, hysteresis, and diarrhea [9,10]. Mycotoxins, including aflatoxin B1 (AFB1), ochratoxin A (OTA), and zearalenone, would migrate from moldy feed to the cow, transform to their main metabolites, and present residues in milk [11,12,13,14]. Typically, AFB1 would metabolize to aflatoxin M1 (AFM1) and M2 (AFM2) in the animal [12,14,15]. Additionally, pesticides are a type of contaminant in bovine milk because they may migrate from direct use as disinfectants and as veterinary drugs to control ectoparasites and insect pests in cow breeding, or indirectly from their migration from contaminated feed and forage [16,17,18].

The residues of these contaminants can cause toxic effects in animals and humans and therefore attract global concern. For example, penicillin can result in allergic reactions in sensitive children or senior citizens; AFM1 and OTA have been defined as potential carcinogens by the International Agency for Research on Cancer (IARC) [14]. Thus, many countries and organizations have established maximum residue limits (MRLs) for veterinary drugs, mycotoxins, and pesticides in bovine milk. As a sample, MRLs of AFM1 in bovine milk were set to 0.05 μg/kg and 0.5 μg/kg by the European Union (EU) [19] and China [20], respectively.

To simultaneously analyze and monitor the residues of different contaminants in milk, various methods have been reported using different analytical techniques such as high-performance liquid chromatography (HPLC) [4,15,17,21,22], gas chromatography/mass spectrometry (GC/MS) [1,23], gas chromatography–tandem mass spectrometry (GC-MS/MS) [2,24], liquid chromatography-mass spectrometry (LC-MS) [25], and liquid chromatography–tandem mass spectrometry (LC-MS/MS) [2,26], liquid chromatography coupled to time-of-flight mass spectrometry (LC-QTOF-MS) [10,27,28,29,30], or orbitrap mass spectrometry [9,12,31,32,33]. LC-MS/MS has more extensive applications because of its good qualitative and quantitative determination ability, and one single method can cover different targeted contaminants, including veterinary drugs [5,6,7,26,28,34,35,36,37,38], mycotoxins [11,14,39,40,41], and pesticides [42]. However, to our knowledge, the contaminants in milk could be not only veterinary drugs or mycotoxins; they could be a mixture.

As an effective instrument to obtain more mass spectral information of the targeted contaminants, ultrahigh-performance liquid chromatography–hybrid quadrupole–linear ion trap mass spectrometry (UHPLC-Qtrap-MS) can use multiple reaction monitoring (MRM)–information-dependent acquisition (IDA)–enhanced product ion (EPI) to simultaneously obtain quantitative and qualitative results in one analysis [43]. This instrument showed more powerful analytical ability than the traditional triple quadrupole mass spectrometer and has already been applied in the determination of amantadine and rimantadine in feed [44].

To detect different types of contaminants in bovine milk more effectively, some more powerful methods need to be developed to simultaneously screen and analyze veterinary drugs, mycotoxins, and pesticides. Thus, this study first employed UHPLC-Qtrap-MS to establish and validate a multiclass and multiresidue method for the simultaneous quantitative and qualitative confirmation of 209 contaminants in bovine milk. Then, the developed method was applied to analyze 140 real milk samples that were collected from supermarkets and dairy farms in China. Finally, the analytical results using LC-MS/MS in the literature since 2014 were collected and comparatively discussed, and the key contaminants that need to be considered in different countries were refined.

## 2. Materials and Methods

### 2.1. Chemicals and Reagents

Veterinary drugs, pesticide, and mycotoxin standards were obtained from Alta Technology (Tianjin, China) with an available purity > 95%. Individual stock solutions at concentrations near 1000 mg/L were prepared in acetonitrile (ACN), methanol (MeOH), or ACN with 50% water (H_2_O) respectively according to their solubility. The mixture of working standard groups was prepared by diluting the stock solutions with ACN. HPLC- and LC/MS-grade solvents used in the study were purchased from Merck (Darmstadt, Germany). Acetic acid (AA), formic acid (FA), and ammonium formate (NH_4_FA) were purchased from Fisher Scientific (Fair Lawn, NJ, USA). Ultrapure water (H_2_O) was prepared from a Milli-Q system (Millipore, Billerica, MA, USA). For the dispersive solid-phase extraction (d-SPE) steps, anhydrous magnesium sulfate (MgSO_4_), primary secondary amine (PSA), octadecyl silane (C18), and EMR-Lipid (EMR) were obtained from Agilent Technologies (Santa Clara, CA, USA), and zirconium dioxide (ZrO_2_) was purchased from Sinopharm (Beijing, China).

### 2.2. Apparatus and Chromatographic Conditions

Sample analysis was achieved in a UHPLC (Exion LC, SCIEX, Wilmington, DE, USA) coupled to a triple quadrupole linear ion trap mass spectrometer (Qtrap 6500+, SCIEX, Wilmington, DE, USA) system. The analytical column setup was a ZORBAX RRHD Eclipse Plus C18 (3.0 mm × 150 mm, 1.8 μm, Agilent Technologies, Santa Clara, CA, USA) with a flow rate of 0.4 mL/min at 40 °C. The mobile phase A was H_2_O, B was MeOH, and both phases included 5 mmol/L NH_4_FA and 0.1% FA. The elution gradient started with 2% B for 0.5 min, which was followed by 25% B, 3 min; 30% B, 6 min; 35% B, 9.5 min; 80% B, 15 min; 100% B, 18 min; 100% B, 22 min; 2% B, 22.1 min; and 2% B, which was maintained for 4 min. The sample injection volume was 2 μL. The parameters of the ion source were as follows: source temperature was set at 500 °C, curtain gas was 30 psi, and GAS 1 and GAS 2 were 55 psi and 50 psi, respectively. In this experiment, MRM analyses were carried out with both positive and negative mode for regular detection. For confirming the positive samples, the MRM-IDA-EPI mode was used. The MRM parameters, declustering potential (DP), and collision energy (CE) are listed in Table S1.

### 2.3. Sample Preparation

A modified QuEChERS sample preparation method was used. The sample preparation procedure began with a comparison of extraction solutions by ACN with different concentrations of AA and also FA. Then, the cleanup step was evaluated by different sorbents including PSA, C18, EMR, and ZrO_2_, and each of them was tested pairing with MgSO_4_. The final steps followed: 5 g milk samples were weighed into 50 mL centrifuge tubes, spiked with different drug standard groups and allowed to stand for 30 min at room temperature. During the extraction process, 10 mL of ACN with 2% FA were added into samples and mixed on an automatic vortexer for 1 min. To stratify the organic phase and water, 2 g NaCl were added to each tube. After 1 min of vortexing and centrifuging (4500 r/min for 5 min), 1 mL extracts (upper layer) were transferred to 5 mL centrifuge tubes containing 50 mg C18, which were shaken for 30 s. The tubes were centrifuged at 4500 r/min for 1 min and filtered with 0.22 μm membranes before injecting into the UHPLC-MS/MS.

### 2.4. Sample Collection and Analysis

This study was performed with 140 milk samples collected in China, including 99 samples obtained from dairy farms and 41 samples bought from supermarkets. In the supermarket samples, 12 samples were from domestic production, and 29 samples were from imports, according to their labels, including Australia, New Zealand, Germany, and America. All these samples were prepared and analyzed with the developed method, and the results of contaminants were comparatively analyzed with different sources and positive reports in the previous literature.

## 3. Results and Discussion

### 3.1. Optimization of Sample Preparation

The normal optimization of the extraction procedure is usually performed by investigating recovery. In this study, the extraction solution was first optimized by the matrix effects (MEs) of 209 chemicals from various concentrations of AA in ACN. MEs are problematic and important in residue analysis when using MS detection. The slopes of calibration curves of matrix-matched standards and solvent-based standards were compared to estimate the ME. The ME values were divided into three parts to describe the effects on analytes. If the ME values fell between 0.8 and 1.2, the matrix did not exert very strong effects on chemicals. When the ME values were higher than 1.2 or lower than 0.8, the MEs were considered enhancement and suppression, respectively. In this study, weak effects on analytes would be the primary condition for choosing the extraction solution. Therefore, the increase in quality of analytes of value between 0.8 and 1.2 reflects the weak effects of the matrices.

**Figure 1:**
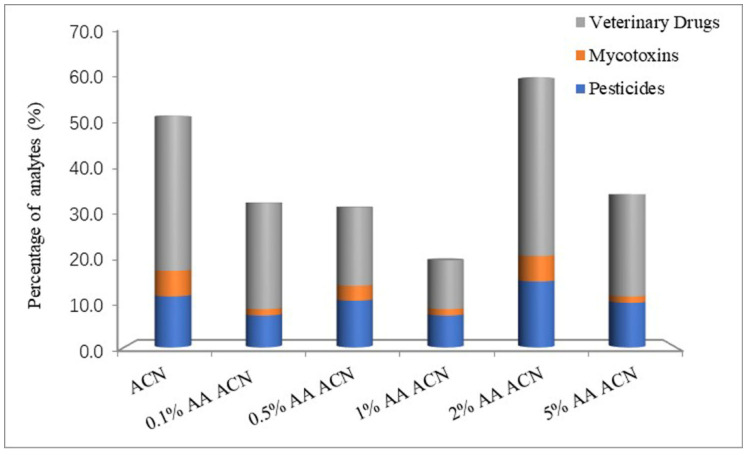
Comparison of ME values between 0.8 and 1.2 from the series of AA in ACN extraction solutions.

**Figure 2:**
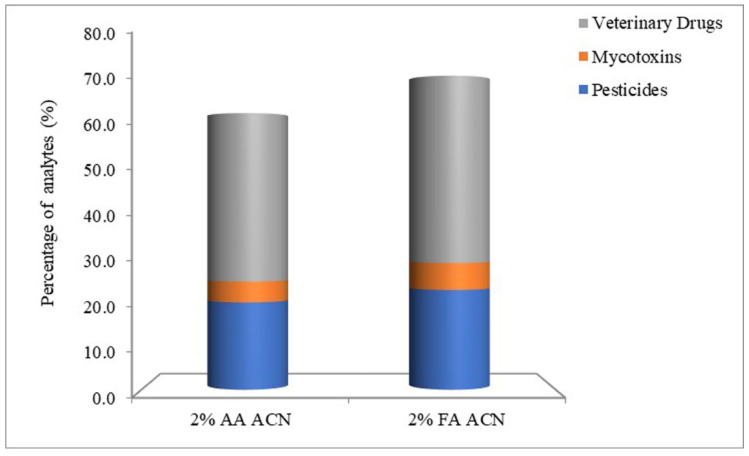
Comparison of ME values between 0.8 and 1.2 by extraction of 2% AA ACN and 2% FA ACN.

The milk samples were extracted by ACN with 0%, 0.1%, 0.5%, 1%, 2% and 5% AA. Before the cleaning procedure, extraction solutions were filtered similarly to the matrix solutions to dilute the standards. The matrix curves of six extraction solutions were compared with solvent curves respectively to calculate the ME. Figure 1 shows an obvious effect on MEs by different percentages of AA. With the increase in acidity, the number of MEs between 0.8 and 1.2 does not present a regular change. ACN with 2% AA shows the best results in both pesticides and veterinary drugs. For mycotoxins, 2% AA ACN is similar to ACN. To analyze the difference between AA and FA, 2% FA ACN was used as an extraction solution to compare with 2% AA ACN. From Figure 2, the number of MEs between 0.8 and 1.2 from 2% FA ACN is higher than with 2% AA ACN for all classes of analytes. Thus, the best extraction solution for this study was 2% FA ACN from the results of the ME evaluation.

**Figure 3:**
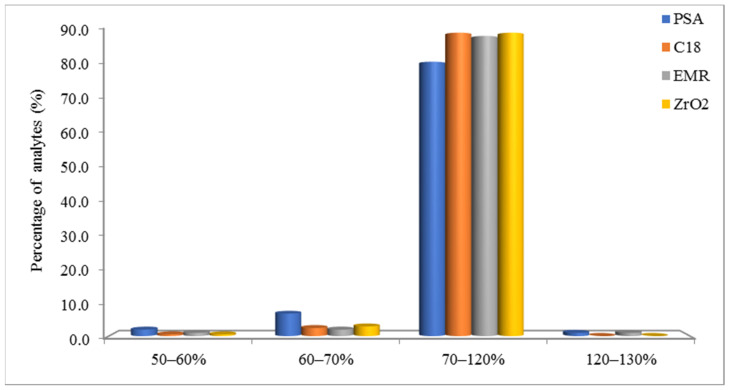
Comparison of recovery by different absorbents.

A d-SPE cleanup step was evaluated after extraction by 2% FA ACN. Extracts (1 mL of the upper layer) were transferred to 5 mL centrifuge tubes with sorbents. The initial cleanup step was tested with four sorbents, PSA, C18, EMR and ZrO_2_, and 50 mg of each of them was mixed with 150 mg MgSO_4_. However, the MgSO_4_ showed a strong adsorbability for sulfonamides and tetracyclines. Therefore, the next step was the evaluation of every single adsorbent. The range of recovery was separated into four stages, and each stage calculated the percentages of analytes. From Figure 3, C18 and ZrO_2_ outperformed the other two sorbents, because we prefer more chemical recoveries falling in the range of 70% to 120%, although individual compounds were still outside the range. C18 and ZrO_2_ showed very similar results on recovery. However, ZrO_2_ had not been used as commonly as C18 in the residual analysis. Therefore, C18 was used in the d-SPE cleanup step. To compare with the classic column SPE method, d-SPE removes the procedures of activating agent and elution, which provided a more efficient cleaning procedure.

### 3.2. Optimization of UHPLC-Qtrap-MS

Mass spectrometric parameters were optimized to maximize sensitivity. To increase the dwell time of each transition, the scheduled MRM model was used, and the chromatogram of each chemical was maximized split on the column to reduce the number of transitions at each time point. Then, the mobile phase compositions were evaluated. We found that NH_4_FA and FA would show an impact on sensitivity. Thus, low amounts of NH_4_FA and FA were added to both aqueous and organic (MeOH) phases. FA (0.05%, 0.1%, 0.2%) was added to the 5 mmol/L NH_4_FA-based mobile phase to evaluate the impact on the MS signal. However, it was very difficult to demonstrate a signal increase for every chemical. The result showed that the mobile phase with 0.1% FA and 5 mmol/L NH_4_FA was the best choice. Under these conditions, most of the chemical signals increased. At the same time, there was a slight diminishing in negative ion data acquisition, but the sensitivity was still strong enough.

### 3.3. Method Validation

The parameters for method validation included linearity, detection limits (LODs), quantification limits (LOQs), precision, and accuracy. Calibration curves were obtained for concentrations of 0.25, 0.5, 2.5, 5, and 12.5 μg/L for veterinary drugs and 0.05, 0.25, 0.5, 2.5, and 5 μg/L for mycotoxins and pesticides. The standards were prepared in ACN and matrix solvent, respectively. Both solvent and matrix curves exhibited excellent linearity over the working range, and *R*^2^ values were ≥0.99. LODs for every chemical ranged from 0.01 to 1 μg/kg, and LOQs ranged from 0.05 to 5 μg/kg. All the limits were under or equal to the MRL that was based mainly on the regulations from China [20] and EU [19] if the chemicals had no regulatory levels in China (Table S1).

Accuracy was evaluated at spiked concentrations of 0.5, 1, 5, 10, and 25 μg/kg for veterinary drugs, and 0.1, 0.5, 1, 5, and 10 μg/kg for mycotoxins and pesticides in milk. The average recoveries of 209 chemicals ranged from 51.2 to 129.76%. Salbutamol, cefixime, and epi-chlortetracycline showed lower recoveries (between 51.2% and 65.3%) at different spiking levels. Furthermore, because of the lower MRL, aflatoxin M1, diclofenac, and clenbuterol were increased for the testing level. The MRL of aflatoxin M1 is 0.5 μg/kg in China but 0.05 μg/kg in Europe. Therefore, we increased the concentration of 0.05 μg/kg spiked in milk samples. The diclofenac increased the spiking concentration to 0.1 μg/kg, which is the MRL in Europe. Clenbuterol is prohibited in China, but the MRL is 0.05 μg/kg in Europe. Therefore, these concentration levels were also spiked in samples to test recovery. Fortunately, these three analytes had a very perfect sensitivity at the lower concentration, and their recoveries ranged between 110% and 118%. Relative standard deviation (RSD) values were used to express precision. From the results, the RSDs of all analytes were from 0.82% to 19.86%, including salbutamol, cefixime, and epi-chlortetracycline (Table S1).

### 3.4. Application of the Developed Method to Milk Samples

The real milk samples were collected and analyzed with the developed method, which demonstrated good applicability for the screening, quantitation, and confirmation of three types of contaminants in milk. As Table 1 shows, contaminants including veterinary drugs (sulfamethazine and cloxacillin), mycotoxins (aflatoxin M1), and pesticides (fipronil sulfone, imidacloprid, and acetamiprid) were found in real milk samples.

Comparatively, veterinary drugs showed a higher detection rate than mycotoxins and pesticides. Sulfamethazine and cloxacillin were often used to prevent bovine mastitis but are prohibited in the lactation stages. Cloxacillin can be more widely applied in China than sulfamethazine, because its detection rate (8.85%) was higher than that of sulfamethazine (0.71%). Additionally, cloxacillin was detected in the samples from the supermarket and the dairy farm simultaneously with three samples exceeding the values of the Chinese MRL (30 μg/kg), indicating that the residues of cloxacillin from the farm may sustainably exist in the final product.

The lower concentrations (0.17 and 0.24 μg/kg) of AFM1 were found in milk samples that were under the MRL (0.5 μg/kg) set by China. Both of the two positive samples came from the dairy farm, and the possible source may be the migration and metabolism of AFB1 from the moldy feed in cow breeding that can produce mycotoxins, especially under conditions of higher temperature and humidity.

Fipronil sulfone (0.08 μg/kg) is the main metabolite of fipronil that resulted in an outbreak of egg contamination in Europe in 2017 because of the use of fipronil in a disinfectant. Fipronil is a broad-spectrum insecticide, which is used as the active ingredient in field pest control for corn and flea control products. The MRL in milk is 8 μg/kg in the EU, which is the sum of fipronil and the sulfone metabolite. Imidacloprid and acetamiprid are both insecticides that are widely used for pest control in agriculture. The MRLs of imidacloprid and acetamiprid in milk are 100 μg/kg and 200 μg/kg, respectively, in the EU. All the values detected in milk samples were under the MRLs. However, these results indicate that the pesticides may be directly used at dairy farms to control the harmful parasites and flies or migrate from the contaminated forage or feeds made from crops on which the pesticides were used.

The higher detection rates of contaminants were observed in milk samples from dairy farms compared with the supermarkets, indicating that the farm is a key control point for pollution prevention and quality control of milk. Additionally, both the domestic and imported products contained the residues of cloxacillin above MRL, suggesting that its contamination is a joint problem in different countries.

### 3.5. Qualitative and Quantitative Confirmation

The positive samples were further qualitatively confirmed using UHPLC-Qtrap-MS in the MRM-IDA-EPI mode, which can obtain quantitative and qualitative results based on simultaneous scans of MRMs and second product ions in one injection. Usually, the MRMs together with retention time matching could confirm a finding. However, the effects of the matrix and the structural similarity of compounds could bring interference. Thus, the EPI mass spectra will give further qualitative confirming information. Collision energy had a very strong impact on the product ion information. Thus, a collision energy spread (CES) of 15 V was used to obtain the average fragment ion spectrum with different CEs of 15 V, 30 V, and 45 V. The scan rate of EPI was set on 10,000 Da/s from *m*/*z* 50 to 200. The solvent standards of targeted contaminants with different concentrations were analyzed first, and all the standard spectra were imported to the database library and then selected as references. The matching correlation of molecular and fragment ion data from the library is typically expressed as the purity fit value, and 70% had been set by the instrument manufacturer as an appropriate threshold for the positive identification of the analytes in samples [43].

**Figure 4:**
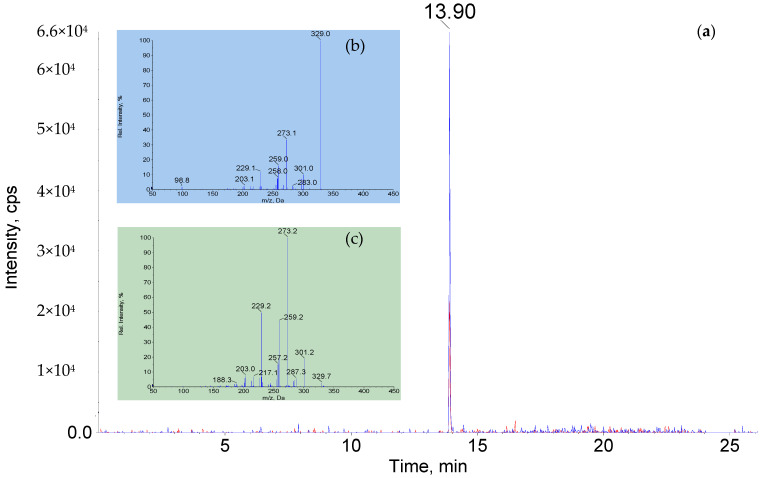
(**a**) Typical MRM chromatogram of aflatoxin M1 in bovine milk; (**b**) Product ion spectrums of aflatoxin M1 in bovine milk; (**c**) Product ion spectrums of aflatoxin M1 in standard product ion spectrums in database library.

The purity fit values were observed at >83% for sulfamethazine, cloxacillin, and aflatoxin M1 and >76% for fipronil sulfone, imidacloprid, and acetamiprid. Typical comparative MRM chromatograms and product ion spectra of aflatoxin M1 in milk and in standard solution are shown in Figure 4. The higher similarity of fragment ions from EPI was observed between the reference and the unknown spectra from real samples, and thus, we can more effectively improve the qualitative credibility and reliability of the method compared to the traditional MRM detection.

### 3.6. Comparative Analysis of Contaminants in Milk

The reported results for veterinary drugs, mycotoxins, and pesticides detected in bovine milk using LC-MS/MS in the literature since 2014 and relevant MRLs are listed in Table 2. As was shown, there are 51 contaminants and relevant metabolites including 39 veterinary drugs, 6 pesticides, and 6 mycotoxins that were reported in bovine milk in the literature and in the present study. Veterinary drugs had the highest percentage (76.47%) among the three types of contaminants.

In total, 39 veterinary drugs were found in milk with low concentrations compared to relevant MRLs. Among these drugs, fenbendazole sulfone and desacetyl cephapirin were detected as metabolites of fenbendazole and cephapirin, respectively. The most frequently detected classification was beta-lactams (15 drugs, accounting for 37.5%) compared to fluoroquinolones (five drugs, accounting for 12.5%) and sulfonamides (four drugs, accounting for 10%). Nine drugs and six drugs were cephalosporins and penicillins (beta-lactams), respectively. Penicillin G and cloxacillin (beta-lactams), enrofloxacin and ciprofloxacin (fluoroquinolones), and sulfamerazine (sulfonamide) showed more frequent detection compared to other drugs, indicating that these drugs may be more widely applied in these countries. Cloxacillin and sulfamethazine were also detected in this study, which further proved their wide application. Penicillin G, cefazolin, ampicillin, and cloxacillin are worthy of attention because they contained high concentrations over the MRL in some milk samples. The main reason may be the nonstandard and illegal uses of these veterinary drugs in cow breeding.

Of all targeted contaminants, aflatoxin was the most frequently detected. Especially, AFM1 was detected more frequently than AFB1 because most of the AFB1 would metabolize to AFM1 and AFM2 in bovines, and some of them would migrate into the milk. AFM1 is the main metabolite and was often detected in bovine milk in different countries, including in this study. Additionally, alpha-zearalenol is a metabolite of zearalenone, and residual amounts of both were found in milk. Individual cases of exceeding MRL were observed for AFM1 in the literature [12]. Our detection results showed two samples with lower AFM1 residues from dairy farms.

All pesticides from the list including fipronil, acetamiprid, clothianidin, imidacloprid, thiacloprid, and thiamethoxam are insecticides, and no fungicides and herbicides were found in milk. According to these, it can be suggested that the insecticides were more likely used at dairy farms for hygienic purposes, and no obvious residues of fungicides and herbicides would be produced in milk because of indirect contamination from feed and forage. Both imidacloprid and acetamiprid were found in Switzerland [38]. The residual concentrations of these two pesticides were much lower than in the real samples analyzed in this study. Our data showed that the samples with acetamiprid residues came from a dairy farm in China, and the sample with imidacloprid residues came from the supermarket, which was labelled as an import from Germany. However, the concentrations of these compounds were all far below the MRLs in China and the EU.

In conclusion, beta-lactams, fluoroquinolones, sulfonamides, and aflatoxins were the main contaminants in bovine milk according to the present literature. Penicillin G, cloxacillin, enrofloxacin, ciprofloxacin, sulfamerazine, and aflatoxin M1 should be given more attention for monitoring and control. The nonstandard and illegal uses of veterinary drugs and pesticides are the main reason to cause their residues to be observed in milk. Additionally, the dairy farm was the main source for these contaminants and should have strict regulatory management in cow breeding.

## 4. Conclusions

The present method consists of simple sample preparation and a simultaneous screening and confirmatory analysis with UHPLC-QTRAP-MS using the mode of MRM-IDA-EPI, which shows a good quantitative and qualitative ability at the same time. It is also a highly efficient analytical method with advantages of saving time and costs on sample analysis. The method has been validated for achieving 209 contaminants including veterinary drugs, mycotoxins, and pesticides in bovine milk. After applying it to the milk samples obtained from dairy farms and supermarkets, the low concentration of cloxacillin, aflatoxin M1, acetamiprid, and fipronil sulfone were found. Compared to the results from the literature, some contaminants need to receive more attention regarding their monitoring and control.

## Figures and Tables

**Table 1:** The analytical results from 140 milk samples.

No.	Contaminant	Concentration (μg/kg)	MRL (μg/kg)	Detection Rate (%)	Source
1	sulfamethazine	1.79	100 (China)	0.71	supermarket
2	cloxacillin	9.20–36.14	30 (China)	3.57	dairy farm
		7.12–69.70		4.29	supermarket
3	aflatoxin M1	0.17, 0.24	0.5 (China)	1.43	dairy farm
4	fipronil sulfone	0.08	8 (EU) ^1^	0.71	dairy farm
5	imidacloprid	6.24	100 (EU)	0.71	supermarket
6	acetamiprid	2.36–12.24	200 (China)	2.14	dairy farm

^1^ Sum of fipronil and sulfone metabolite, expressed as fipronil.

**Table 2:** The contaminant concentration in milk from the different countries from the literature.

No.	Country	Contaminant	Concentration	MRL	Reference
			(μg/kg)	(μg/kg)	
1	China	AFB1	0.8, 1.3		[45]
2	China	AFM1	0.046–0.237	0.5 (China)	[14]
		ochratoxin A	0.058–0.084		
		zearalenone	0.028–0.046		
		α-zearalenol	0.045–0.074		
3	China	hydrocortisone	0.14–1.02	10 (China)	[46]
4	China	zearalenone	0.085		[47]
5	China	cefminox	5.6		[48]
		cefradine	2.8		
6	China	sulfadimethoxine	21.5	100 (China) *	[49]
		sulfamerazine	10.6	100 (China) *	
		enrofloxacin	15.3	100 (China)	
7	China	sulfadiazine	0.3–9.7	100 (China) *	[31]
		sulfamerazine	4.9–7.4	100 (China) *	
8	China	AFM1	19.2	0.5 (China)	[12]
		AFM2	3.91		
9	India	sulfamerazine	1.2–18.2		[2]
		cloxacillin			
		ciprofloxacin			
		enrofloxacin			
		oxytetracycline			
		fenbendazole			
		oxfendazole			
10	Italy	albendazole	0.11–0.52	100 (EU)	[50]
		fenbendazole sulfone	0.32–1.21	10 (EU)	
		oxfendazole	0.25–1.83	10 (EU)	
		febantel	0.13–0.28		
11	Italy	amoxicillin	1.26	4 (EU)	[51]
		marbofloxacin	0.52–0.91	100 (EU)	
12	Italy	penicillin G	1.51–236	4 (EU)	[35]
		dihydrostreptomycin	5.85–197	200 (EU)	
13	Brazil	tilmicosin	>MRL	4 (EU)	[28]
		cloxacillin		30 (EU)	
		ceftiofur		100 (EU)	
14	Switzerland	thiamethoxam	0.002–0.0085		[42]
		clothianidin	0.004–0.013		
		imidacloprid	0.0025–0.0065	100 (EU)	
		acetamiprid	0.0005–0.0095	200 (EU)	
		thiacloprid	0.0001–0.0002		
15	Brazil	AFM1	0.16–0.48	0.5 (Brazil)	[16]
16	Spain	enrofloxacin	9.3–15.6	100 (EU)	[5]
		ciprofloxacin	5.6–32.4	100 (EU)	
		marbofloxacin	4.4, 11.3	100 (EU)	
		amoxicillin	2.1–18.6	4 (EU)	
		penicillin G	2.4–5.3	4 (EU)	
		cephalonium	4.6		
		dicloxacillin	3.5	30 (EU)	
17	Malaysia	AFM1	0.004, 0.01		[15]
18	China	ochratoxin A	0.37, 1.59		[39]
		zearalenol	3.25		
		AFB1	0.21, 0.52		
		AFM1	0.01	0.5 (China)	
19	China	ofloxacin	13.1–36	100 (China)	[7]
		enrofloxacin	14.2–24	100 (China)	
		ciprofloxacin	14–44	100 (China)	
20	Spain	danofloxacin	0.7–1.5		[26]
21	Italy	cephapirin	3–6	60 (EU)	[33]
		cefazolin	9–87	50 (EU)	
		oxytetracycline	4	50 (EU)	
		penicillin G	2–10	4 (EU)	
		cefoperazone	4	50 (EU)	
		dicloxacillin	4	30 (EU)	
		desacetyl cephapirin	4, 8	50 (EU)	
		ampicillin	2–7	4 (EU)	
		cloxacillin	2, 32	30 (EU)	
		cefacetrile	10	125 (EU)	
		rifaximin	11	60 (EU)	
		lincomycin	2	150 (EU)	
22	China	phenobarbital	8		[52]
		pentobarbital	10.8		
		amobarbital	12.5		
		secobarbital	7.4		

* Sum of different compounds of the same classification.

## Data Availability

The data shown in this study are contained within the article.

## Supplementary Materials

The following supporting information can be downloaded at https://www.mdpi.com/article/10.3390/foods11030331/s1, Table S1: MRM parameters, recoveries, RSDs, LODs, LOQs, and MRLs of China or EU for 209 contaminants.
