# Supplementary material for: Multiclass Comparative Analysis of Veterinary Drugs, Mycotoxins, and Pesticides in Bovine Milk by Ultrahigh-Performance Liquid Chromatography–Hybrid Quadrupole–Linear Ion Trap Mass Spectrometry
Source: Foods. 2022 Jan 25;11(3):331. doi: 10.3390/foods11030331 (PMC8834082; doi:10.3390/foods11030331)
Supplement: Supplementary file 1 [file foods-11-00331-s001.zip › foods-1549667-supplementary.pdf]

Table S1. MRM parameters, recoveries, RSDs, LODs, LOQs, and MRLs of China or EU for 209 contaminants

No.	compound	RT (min)	precursor （m/z)	product 1 （m/z)	product 2 （m/z)	DP (V)	CE 1 (V)	CE 2 (V)	spiked concentration (μg/kg)	recovery (μg/kg)	RSD (%)	LOD (μg/kg)	LOQ (μg/kg)	MRL (μg/kg)
1	difenoconazole	18.42	406.1	251	337	75	35	20	1,5,10	97.63, 96.32, 87.66	5.22, 1.53, 2.54	0.5	1	5 (EU)
2	imidacloprid	10.20	256.1	209	175	75	20	20	1,5,10	110.78, 105.20, 90.31	3.02, 4.17, 9.71	0.5	1	100 (EU)
3	profenofos	18.87	373	302.9	345.2	75	25	20	0.1,1,10	98.27, 88.10, 82.51	10.37, 3.13, 4.13	0.05	0.1	10 (EU)
4	diflubenzuron	17.68	311	158	141.2	70	20	45	1,5,10	120.80, 117.20, 110.14	6.20, 8.27, 5.51	0.5	1	50 (EU)
5	pyridaben	19.85	365	309	147	55	20	30	0.1,1,10	96.30, 78.64, 70.53	17.38, 4.58, 3.98	0.05	0.1	20 (EU)
6	dichlorvos	14.89	220.9	109	145	60	22	18	1,5,10	102.02, 101.98, 91.90	3.18, 5.15, 2.76	0.5	1	
7	acetamiprid	11.73	223	126	99	70	25	50	0.1,1,10	118.37, 104.47, 88.44	3.58, 2.29, 4.26	0.05	0.1	200 (EU)
8	chlorpyrifos	19.27	350	198	97	75	20	46	0.1,1,10	116.65, 93.61, 81.35	4.45, 3.97, 5.17	0.05	0.1	10 (EU)
9	parathion	17.86	292	236	264	90	19	13	0.1,1,10	110.00, 94.71, 95.70	19.81, 11.34, 5.97	0.05	0.1	50 (EU)
10	carbendazim	8.00	192	160	132	65	25	40	0.1,1,10	102.27, 91.59, 82.34	1.20, 4.09, 4.33	0.05	0.1	50 (EU)[a]
11	pendimethalin	19.36	282.1	212	194	22	15	24	0.1,1,10	102.52, 89.43, 82.52	12.36, 2.80, 3.83	0.05	0.1	10 (EU)
12	diazinon	18.21	305	169	153	65	30	30	0.1,1,10	109.27, 96.47, 88.96	7.3, 6.51, 4.99	0.05	0.1	20 (China)
13	phosalone	18.28	368	182	322	38	20	12	0.1,1,10	105.73, 91.67, 78.60	2.92, 3.55, 5.04	0.05	0.1	10 (EU)
14	tau-fluvalinate	19.70	503	181	208	55	30	20	0.1,1,10	109.54, 78.31, 66.09	13.32, 13.46, 10.57	0.05	0.1	50 (EU)
15	chlorfluazuron	19.40	540	383	158	75	25	25	0.1,1,10	95.47, 87.57, 75.77	12.39, 3.04, 3.40	0.05	0.1	
16	emamectin B1a	18.67	886.4	158	302	40	40	40	0.1,1,10	102.55, 97.49, 90.99	11.82, 2.41, 1.59	0.05	0.1	10 (EU)
17	methamidophos	4.30	142	94	124.9	50	13	15	0.1,1,10	78.95, 76.76, 73.10	8.03, 5.36, 4.12	0.05	0.1	10 (EU)
18	phorate	18.40	261.1	75	199	25	11	10	1,5,10	105.10, 111.97, 97.95	10.65, 13.01, 12.03	0.5	1	
19	phorate sulfone	16.01	293	171	143	50	15	25	1,5,10	117.86, 110.23, 99.67	14.23, 11.99, 11.76	0.5	1	
20	phorate sulfoxide	15.89	277	143	199	70	30	20	1,5,10	106.24, 103.40, 94.18	9.61, 8.92, 5.82	0.5	1	
21	isofenphos-methyl	17.93	273	231	121.1	75	20	35	1,5,10	114.70, 107.14, 97.27	8.94, 10.64, 10.55	0.5	1	

No.	compound	RT (min)	precursor （m/z)	product 1 （m/z)	product 2 （m/z)	DP (V)	CE 1 (V)	CE 2 (V)	spiked concentration (μg/kg)	recovery (μg/kg)	RSD (%)	LOD (μg/kg)	LOQ (μg/kg)	MRL (μg/kg)
22	carbaryl	15.43	202.1	145	127	70	25	40	1,5,10	108.80, 110.96, 105.34	11.19, 6.79, 4.87	0.5	1	50 (EU)
23	fenpropathrin	19.27	350.2	125.1	97.1	55	20	40	0.1,1,10	117.56, 86.12, 75.18	15.22, 3.58, 2.68	0.05	0.1	
24	carbofuran	15.12	222.1	165	123.1	65	20	30	0.1,1,10	118.48, 111.23, 97.94	5.30, 1.69, 6.68	0.05	0.1	＜LOD (China)
25	3-hydroxycarbofuran	11.69	238	181	163	75	20	20	0.1,1,10	111.93, 99.13, 87.16	4.58, 1.93, 5.04	0.05	0.1	
26	dimethoate	11.70	230	125	199	60	25	20	0.1,1,10	113.18, 102.93, 90.89	4.41, 3.40, 3.51	0.05	0.1	
27	bifenthrin	20.29	440.2	181	166	40	18	53	1,5,10	71.50, 64.13, 66.57	9.73, 10.46, 8.64	0.05	0.1	200 (EU)
28	malathion	17.13	331	127	285	40	17	10	1,5,10	123.27, 115.72, 105.22	15.55, 6.63, 11.53	0.5	1	20 (EU)
29	prochloraz	18.23	376.2	308	70.1	65	20	30	1,5,10	108.85, 101.88, 96.12	9.26, 6.78, 8.57	0.5	1	20 (EU)
30	azoxystrobin	16.65	404.1	372	344.1	60	25	35	1,5,10	128.01, 120.91, 92.91	6.97, 3.75, 2.74	0.5	1	
31	pyrimethanil	16.77	200	107	82	70	35	35	1,5,10	93.14, 102.18, 86.86	10.92, 5.04, 9.05	0.5	1	50 (EU)
32	methomyl	7.22	163	106	88	65	20	25	0.1,1,10	112.48, 104.48, 94.13	18.00, 8.96, 4.46	0.05	0.1	
33	chlorbenzuron	17.93	309	156	139	65	25	35	1,5,10	88.35, 85.68, 74.86	3.79, 3.40, 3.83	0.5	1	
34	thiamethoxam	7.90	292	211	181	60	20	30	0.1,1,10	117.69, 104.10, 86.34	3.33, 6.74, 4.57	0.05	0.1	
35	triazophos	17.29	314	162	119.1	60	30	45	1,5,10	111.76, 116.42, 99.74	7.7, 6.88, 4.03	0.5	1	10 (EU)
36	triadimefon	17.24	294	197	225	70	25	20	1,5,10	97.37, 106.06, 90.17	4.85, 4.26, 8.70	0.5	1	100 (EU)[b]
37	fenitrothion	17.28	278	125.1	245.9	95	25	22	1,5,10	112.68, 106.39, 96.86	12.86, 13.07, 7.67	0.5	1	
38	isocarbophos	16.25	273	231	121	40	10	33	1,5,10	106.23, 98.26, 94.74	12.07, 3.00, 3.81	0.5	1	
39	aldicarb	13.90	116.1	89.1	70	38	13	12	1,5,10	105.22, 101.62, 98.15	7.82, 7.74, 7.08	0.5	1	10 (EU)[c]
40	aldicarb sulfone	6.40	223	148	76.1	60	20	25	1,5,10	105.78, 105.91, 92.98	4.83, 6.40, 2.23	0.5	1	
41	aldicarb sulfoxide	5.80	207	132	89	75	20	25	1,5,10	113.8, 100.81, 84.07	12.25, 13.31, 12.90	0.5	1	
42	dimethomorph	17.12	388.1	301	165	70	30	40	0.1,1,10	116.47, 99.79, 92.17	13.61, 6.54, 3.02	0.05	0.1	10 (EU)
43	phoxim	18.17	299.1	77	129	55	35	25	1,5,10	107.08, 100.99, 92.69	3.86, 5.20, 3.04	0.5	1	10 (China)
44	phosmet	16.60	318	160	133	70	25	45	1,5,10	90.87, 93.78, 91.37	5.14, 18.72, 9.26	0.5	1	50 (EU)

No.	compound	RT (min)	precursor （m/z)	product 1 （m/z)	product 2 （m/z)	DP (V)	CE 1 (V)	CE 2 (V)	spiked concentration (μg/kg)	recovery (μg/kg)	RSD (%)	LOD (μg/kg)	LOQ (μg/kg)	MRL (μg/kg)
45	omethoate	5.40	214	182.9	109	70	20	35	1,5,10	120.71, 121.74, 76.38	4.46, 3.77, 9.20	0.5	1	
46	acephate	4.90	184.1	143	125	70	11	23	0.1,1,10	83.81, 82.10, 76.38	7.02, 7.26, 2.92	0.05	0.1	
47	iprodione	17.74	330.1	245	288	40	20	16	0.1,1,10	111.46, 98.37, 89.56	14.78, 7.73, 4.86	0.05	0.1	
48	isocarbophos	17.98	194	95	77	85	15	50	1,5,10	116.08, 110.59, 92.54	11.51, 11.00, 12.59	0.5	1	
49	buprofezin	19.01	305.7	106.3	60	80	45	55	0.1,1,10	111.61, 83.15, 70.90	13.49, 3.35, 2.03	0.05	0.1	
50	tebuconazol	18.22	307.7	70	125	80	45	50	1,5,10	88.94, 85.34, 81.12	5.87, 5.73, 6.18	0.5	1	
51	chlorantraniliprole	16.50	484	453	286	75	35	40	1,5,10	107.86, 99.73, 97.78	14.05, 13.07, 6.17	0.5	1	50 (EU)
52	fipronil	17.60	434.836	329.9	249.8	-55	-25	-35	0.1,1,10	126.86, 103.45, 93.89	2.39, 5.05, 4.37	0.05	0.1	8 (EU)
53	fipronil-sulfone	17.83	451	415.1	281.9	-60	-25	-35	1,5,10	115.15, 111.36, 101.33	2.98, 3.15, 2.20	0.5	1	
54	fipronil-sulfide	17.69	419	383	261.7	-50	-20	-40	1,5,10	110.13, 107.45, 97.75	4.36, 2.36, 1.85	0.5	1	
55	fipronil-desulfiny	17.43	387.1	351.1	281.9	-45	-20	-40	1,5,10	108.5, 99.38, 88.54	5.34, 5.95, 4.67	0.5	1	
56	deoxynivalenol	6.80	297.1	203.1	249.1	50	20	15	1,5,10	82.54, 70.47, 71.02	13.44, 13.16, 15.14	0.5	1	
57	aflatoxin B1	15.00	313.28	285	241.1	95	30	50	0.1,1,10	107.06, 106.13, 86.64	17.16, 19.83, 15.28	0.05	0.1	
58	aflatoxin B2	14.60	315.2	287.1	259.1	95	35	40	0.1,1,10	115.47, 109.9, 89.94	5.93, 6.53, 4.28	0.05	0.1	
59	sterigmatocystin	17.56	325	310	281	85	35	45	0.5,5,10	96.12, 90.00, 80.22	17.02, 13.89, 9.68	0.1	0.5	
60	aflatoxin G1	14.18	329	311.1	243	95	30	35	0.1,1,10	117.35, 110.23, 88.23	6.21, 3.70, 7.46	0.05	0.1	
61	aflatoxin G2	13.64	331.29	245.1	313.1	95	40	35	0.1,1,10	113.29, 105.01, 88.29	11.83, 3.68, 4.43	0.05	0.1	
62	3-acetyl-deoxynivalenol	12.69	339.2	203	213.3	55	25	25	1,5,10	114.27, 91.11, 82.75	13.08, 7.63, 7.85	0.5	1	
63	15-acetyl-deoxynivalenol	12.71	339.3	261	137.1	75	15	20	1,5,10	109.79, 90.22, 80.22	14.8, 6.74, 16.79	0.5	1	
64	ochratoxin B	16.23	370.2	204.9	102.8	70	30	55	0.5,5,10	115.64, 104.77, 99.21	17.35, 9.60, 4.74	0.1	0.5	
65	ochratoxin A	17.10	404	239	358	60	30	20	0.5,5,10	105.92, 93.61, 85.73	16.91, 7.30, 10.43	0.1	0.5	
66	HT-2	16.10	447.3	345.2	285.1	65	25	25	1,5,10	110.35, 86.40, 68.89	11.37, 14.53, 11.44	0.5	1	
67	T-2	16.65	489.31	387.2	245.1	65	30	35	0.5,5,10	112.11, 79.25, 71.32	13.31, 6.09, 11.84	0.1	0.5	

No.	compound	RT (min)	precursor （m/z)	product 1 （m/z)	product 2 （m/z)	DP (V)	CE 1 (V)	CE 2 (V)	spiked concentration (μg/kg)	recovery (μg/kg)	RSD (%)	LOD (μg/kg)	LOQ (μg/kg)	MRL (μg/kg)
68	verruculogen	17.91	534.32	392.2	435.2	65	20	20	0.5,5,10	125.62, 87.22, 83.30	14.90, 8.82, 13.26	0.1	0.5	
69	aflatoxin M1	13.80	329.1	273	259.1	100	34	35	0.05,1,10	108.83, 104.27, 84.29	9.35, 4.86, 2.55	0.01	0.05	0.05 (EU)
70	aflatoxin M2	13.16	331.1	273	259.1	80	33	34	0.1,1,10	122.51, 107.69, 84.32	6.29, 7.68, 4.01	0.05	0.1	
71	zearalenone	17.22	317	175	131	-100	-31	-34	0.5,1,10	103.11, 87.06, 91.04	11.96, 5.75, 4.30	0.1	0.5	
72	medroxyprogesterone acetate	18.13	387.1	327.3	123	96	18	30	0.5,5,25	126.44, 95.24, 81.90	15.84, 3.68, 5.63	0.1	0.5	
73	methyltestosterone	17.69	303.2	109	97	106	30	28	0.5,5,25	117.45, 90.16, 79.82	19.10, 4.34, 7.37	0.1	0.5	
74	testosterone propionate	19.33	345.2	97	109	114	23	29	0.5,5,25	93.50, 80.74, 74.19	8.29, 1.81, 1.49	0.1	0.5	＜LOD (China)
75	chlormadinone acetate	18.05	405.2	309.2	345.2	100	21	17	0.5,5,25	111.18, 98.79, 86.63	17.91, 6.69, 6.02	0.1	0.5	2.5 (EU)
76	boldenone	16.83	287.1	121	135	85	25	19	0.5,5,25	123.70, 91.27, 87.61	3.03, 5.30, 8.35	0.1	0.5	
77	trenbolone	16.70	271	253.1	199	90	27	33	0.5,5,25	124.29, 116.45, 100.34	19.62, 8.10, 2.99	0.1	0.5	＜LOD (China)
78	nadrolone phenylpropionate	19.86	407.2	105.2	257.2	80	36	25	0.5,5,25	87.39, 67.29, 62.25	10.60, 4.57, 5.22	0.1	0.5	
79	glibenclamide	17.49	494.2	369	169	90	18	45	0.5,5,25	125.13, 107.20, 91.76	13.31, 16.17, 13.91	0.1	0.5	
80	glimepiride	17.84	491.2	352	126.2	90	19	34	0.5,5,25	124.66, 113.43, 93.92	8.50, 10.38, 6.93	0.1	0.5	
81	glipizide	16.28	446.2	321.2	103	85	20	62	0.5,5,25	108.89, 92.89, 88.41	13.79, 16.4, 9.67	0.1	0.5	
82	repaglinde	18.71	453.3	230.2	162	100	38	27	0.5,5,25	105.60, 99.52, 86.54	8.67, 3.05, 3.34	0.1	0.5	
83	tolbutamide	15.94	271.1	155	91.1	72	24	45	0.5,5,25	99.04, 99.68, 91.06	9.39, 5.87, 5.87	0.1	0.5	
84	hydrocortisone acetate	18.05	405.3	309.2	327.2	80	25	24	0.5,5,25	114.38, 101.27, 88.90	6.16, 14.51, 3.59	0.1	0.5	
85	tenoxicam	13.36	338.2	121.1	78	80	26	80	0.5,5,25	108.23, 104.13, 92.73	6.11, 2.51, 3.94	0.1	0.5	
86	flufenamic acid	18.52	282.2	264	167	40	24	55	1,5,25	129.85, 102.54, 95.03	10.2, 3.99, 6.27	0.5	1	
87	indoprofen	16.12	282.2	236	218	85	28	43	0.5,5,25	115.04, 105.44, 85.75	10.45, 7.56, 8.80	0.1	0.5	
88	sasapyrine	16.51	259.2	120	119	50	22	22	0.5,5,25	97.91, 103.85, 100.29	11.74, 7.41, 13.46	0.1	0.5	
89	tolfenamic acid	18.99	262.2	244.1	209	40	21	38	0.5,5,25	120.15, 99.20, 92.41	13.73, 12.07, 3.76	0.1	0.5	50 (EU)
90	meloxicam	16.37	352.1	115	141	50	24	26	0.5,5,25	121.60, 108.83, 102.17	16.54, 6.85, 12.72	0.1	0.5	

No.	compound	RT (min)	precursor （m/z)	product 1 （m/z)	product 2 （m/z)	DP (V)	CE 1 (V)	CE 2 (V)	spiked concentration (μg/kg)	recovery (μg/kg)	RSD (%)	LOD (μg/kg)	LOQ (μg/kg)	MRL (μg/kg)
91	flunixin	17.59	297.1	279.1	264	45	32	45	0.5,5,25	109.94, 104.24, 85.47	12.73, 4.77, 6.76	0.1	0.5	
92	mefenamic acid	18.73	242.1	224.1	209	40	21	39	1,5,25	117.64, 100.47, 88.36	8.42, 6.81, 2.16	0.5	1	
93	diclofenac	17.92	296.2	215	250.1	32	26	19	0.1,5,25	114.30, 121.50, 106.19	11.27, 4.60, 2.80	0.05	0.1	0.1 (EU)
94	piroxicam	15.63	332.2	95.1	164	50	23	24	0.5,5,25	102.36, 94.94, 90.86	7.45, 5.24, 3.61	0.1	0.5	
95	nabumetone	17.26	229.1	171.1	128.2	40	23	53	1,5,25	128.05, 124.96, 101.38	8.85, 8.29, 13.08	0.5	1	
96	sulindac	16.52	357.2	233	340	80	65	28	0.5,5,25	119.48, 110.55, 106.20	14.6, 17.17, 7.23	0.1	0.5	
97	tolmetin	16.51	258.2	119.1	91	60	24	51	0.5,5,25	101.84, 103.21, 88.04	6.27, 19.69, 7.48	0.1	0.5	
98	indomethacin	17.95	358.2	139	111	60	27	70	0.5,5,25	126.25, 105.89, 98.75	13.63, 6.73, 2.83	0.1	0.5	
99	sulfadiazine	5.50	251.1	156	92	40	22	38	5,10,25	121.63, 106.57, 87.04	4.56, 4.41, 7.00	1	5	100 (China)
100	sulfathiazole	5.70	256	156	108	40	22	32	0.5,5,25	125.92, 114.7, 88.71	11.28, 10.84, 4.08	0.1	0.5	
101	sulfapyridine	6.20	250.1	156.1	108	40	23	32	0.5,5,25	125.81, 108.28, 85.86	3.83, 3.95, 4.13	0.1	0.5	
102	sulfamerazine	6.60	265.2	156.1	172.1	82	25	25	0.5,5,25	119.18, 110.64, 90.84	4.03, 3.69, 5.27	0.1	0.5	
103	sulfamethazine	8.00	279.11	186.1	156	60	23	27	0.5,5,25	118.21, 111.44, 91.45	3.80, 3.54, 4.68	0.1	0.5	
104	sulfamonomethoxine	8.25	281.1	156	126.1	75	25	30	0.5,5,25	119.22, 112.84, 96.05	4.48, 6.31, 4.67	0.1	0.5	
105	sulfamethizole	7.53	271	156.1	108	65	21	36	0.5,5,25	117.77, 106.40, 85.41	2.56, 7.20, 4.81	0.1	0.5	
106	sulfameter	8.30	281.1	156.1	108.1	70	25	35	0.5,5,25	109.08, 104.07, 87.36	2.51, 3.04, 6.76	0.1	0.5	
107	sulfachloropyridazine	9.00	285.1	156	108.1	65	22	37	0.5,5,25	119.43, 113.44, 90.96	3.20, 2.41, 3.52	0.1	0.5	
108	sulfamethoxypyridazine	8.26	281	156	126.1	75	25	27	0.5,5,25	119.29, 110.47, 92.11	2.97, 3.66, 3.84	0.1	0.5	
109	sulfadoxine	10.21	311.11	156.1	108.2	70	30	37	0.5,5,25	114.57, 104.45, 91.21	3.28, 5.33, 5.54	0.1	0.5	
110	sulfadimethoxine	13.21	311.1	156.1	218	70	28	28	0.5,5,25	107.58, 107.5, 87.96	3.29, 4.99, 5.25	0.1	0.5	
111	sulfamethoxazole	9.30	254.1	156	108	65	22	36	1,5,25	128.65, 114.74, 96.49	2.88, 4.20, 4.74	0.5	1	
112	sulfisoxazole	10.20	268.1	156.1	113.2	82	22	25	0.5,5,25	125.23, 116.11, 90.60	5.52, 2.44, 8.94	0.1	0.5	
113	sulfabenzamide	11.30	277.1	156	108	60	19	32	0.5,5,25	115.91, 105.39, 88.04	1.48, 2.10, 3.99	0.1	0.5	

No.	compound	RT (min)	precursor （m/z)	product 1 （m/z)	product 2 （m/z)	DP (V)	CE 1 (V)	CE 2 (V)	spiked concentration (μg/kg)	recovery (μg/kg)	RSD (%)	LOD (μg/kg)	LOQ (μg/kg)	MRL (μg/kg)
114	sulfaquinoxaline	13.63	301.1	156	108	80	24	36	0.5,5,25	112.93, 108.35, 92.85	7.83, 3.86, 2.97	0.1	0.5	
115	sulphacetamide	3.40	215	156	108	52	17	29	0.5,5,25	78.18, 93.55, 71.40	9.66, 5.03, 7.08	0.1	0.5	
116	trimethoprim	7.20	291.1	230.1	123.1	95	33	34	0.5,5,25	99.00, 95.26, 85.08	1.22, 4.93, 6.07	0.1	0.5	50 (China)
117	sulfaphenazole	12.54	315	156	108	90	27	40	0.5,5,25	118.02, 107.62, 93.59	5.16, 6.25, 2.82	0.1	0.5	
118	midazolam	15.02	326.1	291.1	249	110	37	49	0.5,5,25	104.85, 102.69, 92.19	5.13, 5.65, 5.04	0.1	0.5	
119	alprazolam	16.35	309.1	281.1	205	100	39	55	0.5,5,25	101.75, 98.64, 83.07	11.12, 11.55, 6.86	0.1	0.5	
120	clonazepam	15.73	316.1	270	214	90	35	52	0.5,5,25	119.06, 107.77, 99.26	2.34, 4.15, 6.71	0.1	0.5	
121	diazepam	17.17	285.1	193	154	80	40	36	0.5,5,25	104.43, 97.95, 92.13	6.73, 1.90, 5.86	0.1	0.5	＜LOD (China)
122	lorazepam	16.26	321.1	275.1	229.1	80	30	41	0.5,5,25	124.66, 106.18, 92.27	13.73, 11.90, 10.53	0.1	0.5	
123	triazolam	16.22	343.1	308.1	239	100	36	55	0.5,5,25	92.56, 91.40, 89.59	13.34, 8.04, 11.99	0.1	0.5	
124	oxazepam	16.32	287.1	241	269	90	32	21	0.5,5,25	110.45, 105.14, 89.12	10.45, 5.51, 5.13	0.1	0.5	
125	nitrazepam	16.12	282.2	236.1	180	80	34	50	0.5,5,25	103.40, 97.12, 85.76	4.37, 10.73, 5.01	0.1	0.5	
126	hydroxy metronidazole	4.70	188.2	123	126	50	19	23	0.5,5,25	74.86, 62.71, 74.33	10.62, 7.02, 14.65	0.1	0.5	
127	metronidazole	5.92	172.2	127.9	82	50	20	37	0.5,5,25	108.62, 90.73, 84.02	6.28, 2.66, 7.72	0.1	0.5	
128	dimetridazole	6.72	142.2	96	81	65	21	36	5,10,25	89.19, 92.83, 81.64	5.52, 4.36, 5.52	1	5	＜LOD (China)
129	ronidazole	5.73	201.2	140	55	50	15	27	0.5,5,25	119.48, 100.70, 84.63	4.47, 4.20, 9.67	0.1	0.5	＜LOD (China)
130	5-nitrobenzimidazole	9.64	164.2	118	90.9	80	27	46	0.5,5,25	102.93, 86.95, 80.59	4.49, 2.49, 7.30	0.1	0.5	
131	ipronidazole	12.45	170.3	124	109	50	25	33	0.5,5,25	113.41, 96.29, 88.76	11.53, 4.69, 3.23	0.1	0.5	
132	albendazole	16.77	266.3	234	190.9	90	28	44	0.5,5,25	122.24, 114.84, 100.89	14.52, 6.9, 0.99	0.1	0.5	100 (China)
133	albendazole sulfoxide	13.51	282.1	240	208	70	19	34	0.5,5,25	108.54, 98.91, 88.7	1.74, 1.71, 2.47	0.1	0.5	
134	flubendazole	16.34	314.1	282	123	90	32	48	0.5,5,25	128.28, 117.8, 99.97	11.09, 10.9, 10.31	0.1	0.5	
135	fenbendazole	17.41	300.2	268	158.9	100	30	46	0.5,5,25	127.75, 124.75, 100.32	6.32, 11.60, 5.37	0.1	0.5	
136	oxfendazole	14.75	316.2	159	191.1	70	43	27	0.5,5,25	121.16, 109.17, 95.02	3.42, 2.61, 2.18	0.1	0.5	10 (EU)

No.	compound	RT (min)	precursor （m/z)	product 1 （m/z)	product 2 （m/z)	DP (V)	CE 1 (V)	CE 2 (V)	spiked concentration (μg/kg)	recovery (μg/kg)	RSD (%)	LOD (μg/kg)	LOQ (μg/kg)	MRL (μg/kg)
137	mebendazole	16.05	296.3	264.1	77.1	100	32	76	0.5,5,25	124.58, 108.22, 96.97	13.44, 5.65, 9.41	0.1	0.5	
138	thiabendazole	9.78	202.2	175	130.9	60	37	48	0.5,5,25	96.93, 84.28, 76.89	1.33, 3.54, 4.67	0.1	0.5	100 (China)
139	tinidazole	6.93	248.2	121.2	93	80	21	25	0.5,5,25	129.76, 109.21, 87.42	4.23, 4.28, 4.07	0.1	0.5	
140	ornidazole	9.88	220	128	82	70	23	43	0.5,5,25	124.36, 106.88, 89.03	1.73, 9.21, 6.24	0.1	0.5	
141	ractopamine	9.00	302.2	164.1	107.1	80	23	51	0.5,5,25	104.77, 97.42, 87.50	1.82, 3.40, 6.78	0.1	0.5	＜LOD (China)
142	salbutamol	4.87	240.2	148.1	222.1	70	24	15	0.5,5,25	57.17, 54.95, 54.01	4.44, 6.64, 1.98	0.1	0.5	＜LOD (China)
143	terbutaline	4.77	226.2	152	107.1	70	21	36	0.5,5,25	79.06, 68.83, 60.93	3.98, 6.35, 4.67	0.1	0.5	＜LOD (China)
144	cimaterol	4.66	220	202	160	65	13	22	0.5,5,25	96.80, 91.49, 82.85	13.59, 4.48, 4.46	0.1	0.5	＜LOD (China)
145	clenbuterol	10.46	277	203	168.1	65	21	38	0.05,5,25	112.31, 103.93, 91.28	4.49, 2.64, 5.44	0.01	0.05	＜LOD (China)
146	tulobuterol	12.32	228	154	118	65	21	35	0.5,5,25	112.07, 103.96, 88.14	5.57, 2.14, 4.34	0.1	0.5	＜LOD (China)
147	penbutolol	16.45	292.2	236.2	201	73	23	28	0.5,5,25	116.33, 106.74, 89.38	5.36, 10.19, 4.04	0.1	0.5	＜LOD (China)
148	propanolol	14.47	260.1	116.1	183.1	80	23	24	0.5,5,25	119.11, 112.94, 91.39	4.65, 4.03, 7.35	0.1	0.5	
149	clorprenaline	9.74	214	154.1	118	73	23	34	0.5,5,25	110.32, 99.03, 84.36	11.5, 3.75, 4.11	0.1	0.5	
150	enrofloxacin	9.55	360	316.1	245.1	80	25	35	0.5,5,25	98.58, 98.28, 90.82	3.24, 4.11, 4.91	0.1	0.5	100 (China)
151	pefloxacin	8.16	334.1	316.1	290.2	80	27	25	0.5,5,25	71.33, 93.36, 84.38	16.02, 3.58, 4.00	0.1	0.5	
152	ciprofloxacin	9.00	332.1	288.1	245.1	80	25	33	0.5,5,25	75.42, 77.84, 76.33	14.54, 14.78, 6.35	0.1	0.5	
153	ofloxacin	8.05	362.2	318.1	261.1	80	26	38	0.5,5,25	85.99, 87.33, 80.05	5.16, 1.99, 6.37	0.1	0.5	
154	sarafloxacin	11.10	386	342.3	299	80	25	38	0.5,5,25	94.00, 99.80, 91.06	4.30, 2.77, 3.78	0.1	0.5	
155	enoxacin	8.20	321	303	234	80	24	30	0.5,5,25	52.08, 79.26, 74.65	11.39, 3.92, 5.00	0.1	0.5	
156	lomefloxacin	10.00	352	265	308.1	80	33	28	0.5,5,25	89.32, 91.45, 84.02	4.86, 3.16, 4.93	0.1	0.5	
157	nalidixic acid	15.15	233	215	187	68	18	34	0.5,5,25	99.82, 94.18, 86.97	11.35, 4.02, 2.30	0.1	0.5	
158	oxolinic acid	13.77	262	244.1	216.1	70	26	40	0.5,5,25	109.76, 107.93, 95.84	3.20, 3.99, 7.22	0.1	0.5	
159	flumequin	15.40	262.1	244.1	202.1	77	23	42	0.5,5,25	95.62, 105.00, 95.45	19.20, 6.17, 2.79	0.1	0.5	

No.	compound	RT (min)	precursor （m/z)	product 1 （m/z)	product 2 （m/z)	DP (V)	CE 1 (V)	CE 2 (V)	spiked concentration (μg/kg)	recovery (μg/kg)	RSD (%)	LOD (μg/kg)	LOQ (μg/kg)	MRL (μg/kg)
160	danofloxacin	9.43	358.1	340.1	314.1	77	30	24	0.5,5,25	79.43, 90.53, 80.86	5.78, 4.62, 5.32	0.1	0.5	30 (China)
161	difloxacin	10.64	400.1	356.1	299.1	80	28	41	0.5,5,25	101.91, 96.34, 87.38	6.86, 4.18, 6.57	0.1	0.5	
162	orbifloxacin	10.40	396	352	295.2	80	24	32	0.5,5,25	102.25, 96.48, 86.27	3.55, 5.55, 3.30	0.1	0.5	
163	sparfloxacin	12.63	393	349.2	292	80	30	38	0.5,5,25	96.37, 99.61, 92.34	8.23, 2.22, 2.31	0.1	0.5	
164	fleroxacin	7.54	370	326.1	269.2	80	27	35	0.5,5,25	87.57, 93.25, 88.75	3.86, 6.12, 7.89	0.1	0.5	
165	erythromycin	15.86	734.5	576.4	158	30	26	36	0.5,5,25	60.64, 69.03, 60.66	12.76, 11.69, 6.58	0.1	0.5	40 (China)
166	clindamycin	14.67	425.3	126.1	377.1	50	32	27	0.5,5,25	67.82, 67.18, 62.14	4.20, 3.24, 4.39	0.1	0.5	
167	roxithromycin	16.73	837.6	679.5	158.1	50	30	37	0.5,5,25	104.53, 94.87, 86.41	17.03, 11.79, 10.44	0.1	0.5	
168	josamycin	16.50	828.6	174.1	109	70	38	70	0.5,5,25	89.90, 89.21, 82.14	13.27, 8.38, 3.37	0.1	0.5	
169	tylosin	15.68	916.6	174	772.5	150	47	43	1,5,25	118.03, 96.24, 91.30	9.26, 3.74, 4.39	0.5	1	50 (China)
170	kitasamycin	15.86	772.5	109	215.1	140	84	39	1,5,25	93.57, 99.66, 87.18	19.17, 11.55, 3.87	0.5	1	
171	tilmicosin	14.57	869.5	696.5	174.2	100	52	52	1,5,25	86.22, 134.42, 117.93	5.98, 9.48, 3.96	0.5	1	50 (China)
172	cefixime	8.20	454	285	126	55	23	33	5,10,25	59.82, 51.20, 54.66	12.7, 12.64, 9.46	1	5	
173	cefetamet pivoxyl	15.87	511.95	240.9	125.9	50	25	59	1,5,25	115.73, 107.86, 95.86	8.40, 5.63, 4.86	0.5	1	
174	cefazolin	8.50	454.877	323	155.8	60	15	21	1,5,25	64.05, 95.64, 85.76	12.21, 6.80, 5.41	0.5	1	
175	cefamandole lithium	12.00	463	347	158.1	70	12	23	1,5,25	75.91, 96.41, 94.26	10.9, 6.66, 5.39	0.5	1	
176	cefoperazone sodium	10.20	646.4	143	530.2	70	38	15	1,5,25	118.04, 79.70, 79.26	11.86, 18.38, 10.03	0.5	1	
177	cefotaxime	8.00	456	166.9	125	60	28	70	1,5,25	103.20, 89.22, 75.23	13.86, 7.58, 1.88	0.5	1	
178	tetracycline	9.20	445.1	410.2	427.1	80	24	19	5,10,25	79.05, 82.62, 86.09	17.58, 17.81, 3.67	1	5	100 (China)
179	epi-tetracycline	9.20	445	410.2	427.1	80	24	19	5,10,25	69.21, 68.78, 65.56	9.93, 13.04, 16.98	1	5	
180	oxytetracycline	9.60	461.2	426.2	443.2	80	25	17	5,10,25	66.8, 61.52, 60.38	18.15, 6.80, 10.42	1	5	100 (China)
181	chlortetracycline	13.04	479.1	462	444	80	24	28	5,10,25	83.87, 72.04, 72.11	17.54, 18.68, 12.29	1	5	100 (China)
182	epi-chlortetracycline	12.99	479	462	444	80	24	28	5,10,25	65.33, 58.49, 55.95	10.72, 12.64, 19.08	1	5	

No.	compound	RT (min)	precursor （m/z)	product 1 （m/z)	product 2 （m/z)	DP (V)	CE 1 (V)	CE 2 (V)	spiked concentration (μg/kg)	recovery (μg/kg)	RSD (%)	LOD (μg/kg)	LOQ (μg/kg)	MRL (μg/kg)
183	doxycycline	14.18	445	428.1	154.1	80	24	35	5,10,25	82.68, 101.19, 76.64	17.70, 17.07, 12.68	1	5	
184	clopidol	7.52	192.2	100.9	86.9	80	35	42	1,5,25	75.74, 65.53, 77.01	10.91, 14.70, 3.65	0.5	1	20(China)
185	amantadine	10.10	152.1	135	93	70	25	38	1,5,25	95.65, 85.56, 69.47	7.95, 3.10, 4.75	0.5	1	
186	promethazine	15.39	285	86.2	198.1	80	19	27	1,5,25	115.55, 103.57, 92.97	12.87, 4.03, 4.79	0.5	1	
187	rimantadine	14.62	180.1	163	81	80	21	30	1,5,25	118.72, 100.26, 87.75	15.91, 5.91, 5.49	0.5	1	
188	fenoterol	6.10	304.1	135.1	107.2	100	23	36	1,5,25	96.92, 89.14, 80.57	10.13, 9.23, 11.31	0.5	1	
189	phenylethanolamine A	14.43	345.1	327.3	150.1	90	18	30	1,5,25	123.66, 115.33, 96.44	12.93, 2.96, 4.80	0.5	1	
190	bambuterol	13.27	368.2	294.3	72.2	100	26	37	1,5,25	122.60, 102.54, 90.55	8.19, 4.13, 2.83	0.5	1	
191	mabuterol	12.75	311.1	237.2	202.1	110	24	40	0.5,5,25	114.90, 96.05, 91.06	6.93, 3.60, 3.90	0.1	0.5	
192	cimbuterol	5.74	234	160.1	143	70	21	34	0.5,5,25	124.52, 124.29, 120.35	15.4, 13.69, 4.63	0.1	0.5	
193	brombuterol	12.50	367	293	349.2	90	24	17	0.5,5,25	113.76, 97.45, 92.87	3.23, 4.82, 4.04	0.1	0.5	
194	sulfisomidine	5.56	279.1	124	186.1	60	30	23	0.5,5,25	61.72, 90.62, 80.75	8.14, 4.95, 5.70	0.1	0.5	
195	sulfaguanidine	4.91	215	156	108	50	17	35	0.5,5,25	103.84, 91.23, 76.31	2.84, 6.66, 6.72	0.1	0.5	
196	marbofloxacin	7.27	363.4	320.3	72	45	23	46	0.5,5,25	81.57, 76.57, 71.96	14.43, 8.87, 7.49	0.1	0.5	
197	penicillin G	15.01	335.2	176	160	68	16	19	0.5,5,25	88.27, 80.38, 79.49	10.15, 10.18, 7.75	0.1	0.5	4 (China)
198	penicillin V	15.63	351.2	160	114	74	14	46	0.5,5,25	68.04, 87.85, 88.42	14.91, 19.86, 5.04	0.1	0.5	
199	ampicillin	8.73	350	106	160	65	30	19	0.5,5,25	65.48, 64.67, 63.97	18.57, 11.08, 18.15	0.1	0.5	10 (China)
200	oxacillin	15.67	402.2	160	243	80	19	19	0.5,5,25	102.70, 102.65, 97.45	6.83, 12.87, 7.31	0.1	0.5	30 (China)
201	cloxacillin	15.89	436.1	160	277	76	18	19	0.5,5,25	116.45, 106.11, 101.50	17.41, 12.35, 10.28	0.1	0.5	30 (China)
202	clarithromycin	16.62	748.5	590.4	158	40	29	40	0.5,5,25	103.91, 75.23, 79.65	11.86, 8.02, 7.66	0.1	0.5	
203	oleandomycin	15.08	688.4	158.2	544.3	30	70	42	5,10,25	116.11, 102.75, 105.51	9.32, 5.40, 6.65	1	5	
204	tiamulin	15.53	494.3	192	119.1	80	26	54	0.5,5,25	108.69, 109.47, 99.70	11.67, 6.01, 7.02	0.1	0.5	
205	valnemulin	16.73	565.4	263.4	147	80	22	52	1,5,25	102.15, 94.99, 78.74	17.68, 14.00, 12.27	0.5	1	

No.	compound	RT (min)	precursor (m/z)	product 1 (m/z)	product 2 (m/z)	DP (V)	CE 1 (V)	CE 2 (V)	spiked concentration (μg/kg)	recovery (μg/kg)	RSD (%)	LOD (μg/kg)	LOQ (μg/kg)	MRL (μg/kg)
206	abamectin	20.03	895.5	751.4	449.1	50	60	60	1,5,25	120.87, 87.65, 75.12	5.59, 3.16, 6.29	0.5	1	5 (EU)
207	ivermectin	20.03	897.5	753.2	329.2	50	55	70	5,10,25	85.96, 78.36, 70.82	9.09, 8.12, 5.92	1	5	
208	doramectin	20.41	921.5	353.2	449.1	50	70	60	5,10,25	82.41, 69.48, 65.97	10.57, 12.82, 8.79	1	5	
209	eprinomectin	19.83	936.5	490.1	352.1	55	70	75	1,5,25	109.54, 95.30, 85.57	13.1, 13.62, 10.43	0.5	1	

[a] Sum of carbendazim and benomyl.

[b] Sum of triadimefon and triadimenol.

[c] Sum of aldicarb, aldicarb sulfone and aldicarb sulfoxide.
